# Risk assessment behaviour when eating out in adults with food hypersensitivity

**DOI:** 10.1002/clt2.12336

**Published:** 2024-01-31

**Authors:** Rebecca C. Knibb, Lily Hawkins, Cassandra Screti, M. Hazel Gowland, Mamidipudi Thirumala Krishna, George du Toit, Christina J. Jones

**Affiliations:** ^1^ Aston University ‐ Psychology Birmingham UK; ^2^ Department of Health and Community Sciences Faculty of Health and Life Sciences University of Exeter Exeter UK; ^3^ Allergy Action St Albans UK; ^4^ Institute of Immunology and Immunotherapy University of Birmingham Birmingham UK; ^5^ Department of Allergy and Immunology University Hospitals Birmingham NHS Foundation Trust Birmingham UK; ^6^ Department of Women and Children's Health (Pediatric Allergy) School of Life Course Sciences Faculty of Life Sciences and Medicine King's College London London UK; ^7^ Children's Allergy Service Evelina London Children's Hospital Guy's and St Thomas' Hospital London UK; ^8^ Peter Gorer Department of Immunobiology School of Immunology and Microbial Sciences King's College London London UK; ^9^ School of Psychology Faculty of Health & Medical Sciences University of Surrey Guildford Surrey UK

**Keywords:** adults, coeliac disease, eating out, food allergy, food intolerance

## Abstract

**Background:**

Food hypersensitivity (FHS) management requires daily risk assessments of all food and drinks consumed to prevent unpleasant and potentially fatal adverse reactions. Most research has focussed on food allergy in children and families. Little is known about the impact on adults or those with other FHS, such as food intolerance or coeliac disease. This study assessed differences in practices and risk assessment behaviours when eating out for adults with FHS.

**Methods:**

Adult UK residents (*N* = 930; 820 females, 90 males; 95% White; mean age 50 years [±16.6SD]), with food allergy (18%), food intolerance (23%) coeliac disease (44%) or multiple FHS (15%) completed an online survey.

**Results:**

Adults checked information to identify foods causing a reaction always or most of the time when eating out. However, adults with food intolerance reported checking significantly less often than adults with other FHS (all *p*s < 0.001). Adults reporting more severe FHS, medical rather than self‐diagnosis of FHS, previous anaphylaxis, had called an ambulance or been in hospital due to a reaction checked information significantly more often (all *p*s < 0.001), but were also less confident in the information provided (all *p*s < 0.05). Adults with allergy, coeliac disease or multiple FHS were also less confident in written and verbal information provided than those with food intolerance (*p* < 0.01). The type of FHS, greater perceived severity of FHS and having a medical diagnosis consistently predicted risk assessment behaviours when eating out (all *p*s < 0.001).

**Conclusion:**

Clinicians, patients and the food industry should be aware that the type of FHS, patient‐perceived severity and past experience of reactions affect risk assessment behaviours when eating out. This should be considered when providing clinical advice and emergency plans.

## 1 INTRODUCTION

Food hypersensitivities (FHS), including food allergy, food intolerance and coeliac disease, require daily risk assessments of all food and drinks consumed to prevent unpleasant and potentially fatal adverse reactions. Such vigilance has been shown to have an impact on quality of life (QoL) and mental health, particularly for social events such as eating out.^1^, ^2^ Legislation introduced in 2014^3^ required clearer information on allergens for prepacked foods (such as emphasis by font, style or colour) and mandatory allergen information for non‐prepacked foods, which included food sold in places such as cafes and restaurants. This has been a positive step towards aiding those with FHS to manage their condition and has led to feelings of increased provision and allergy awareness.^4^

However, there are variations amongst those living with FHS regarding the provision of different information available. For example, those seeking to avoid milk feel that they have less information available to them when eating out, compared to those with food allergy looking to avoid nuts.^5^ Additionally, they perceived that their hypersensitivity to milk was seen as less serious and so less provision was provided when eating out.^5^ In comparison, those with severe food allergies have reported attempting conversations with staff when eating out to ensure their safety and alleviate worries; however, they were concerned about appearing ‘fussy’.^6^ Studies on consumer preferences have provided further insights into what provisions parents and adults with different allergens would like to see when eating out. For example, participants with food allergies prefer written information but have more confidence in verbal information from asking staff about allergens when eating out.^7^

Given these differences, it is important to further consider the eating out practices of those needing to avoid a variety of foods, to guide clinicians on advice to give to those living with different FHSs, and to inform policy for the food industry. There is little evidence about the eating out practices of individuals with different FHSs. In particular, there is no research looking at behaviour in those with IgE‐mediated allergy compared to those with food intolerance or coeliac disease, whose concerns or experiences might be different. Additionally, while much of the literature has focussed on the needs of parents and children in managing their FHS and related QoL,^8^, ^9^, ^10^ a relatively unexplored group is adults living with different FHSs. It is plausible that the needs of adults may be different from those of children and parents. The aim of this research was to quantitatively investigate practices and risk assessment behaviours when eating out for adults with food allergy, food intolerance, or coeliac disease in the UK.

## 2 METHODS

### 2.1 Design

The data was collected as part of a large‐scale study (the FOODSENSITIVE study) investigating how adults, parents and children manage their FHS (food allergy, food intolerance, coeliac disease) and how this impacts their QoL. Data were collected using an online survey. The study was approved by the Aston University Ethics Committee (#1678). Informed consent was obtained from all participants.

### 2.2 Recruitment of participants and procedure

Eligibility criteria included adults (≥18 years) living in the UK and having food allergy, food intolerance or coeliac disease. The aim was to recruit an inclusive and diverse sample of participants from the community with a self‐reported medical diagnosis or self‐diagnosis of FHS, given that a belief that one has FHS will impact behaviour, whether this is medically diagnosed or not.^11^ Participants were recruited by adverts from patient organisations including Allergy UK, Anaphylaxis UK, Coeliac UK, the Natasha Allergy Research Foundation, a survey panel administered through Qualtrics XM, as well as through social media by the study team and word of mouth. A link in the advert took participants to an information sheet and consent form. Once consent was completed, participants were given access to the survey, which took approximately 30 min to complete. All materials were hosted online using the Qualtrics online survey platform.

### 2.3 Measures

Demographics such as age, gender, ethnicity, employment status and other long‐term health conditions were collected to characterise the sample. Participants were asked to report all the foods they reacted to and were then asked to report in detail up to three of those foods that they felt had the most impact on their lives. For these foods, they were asked about symptoms, time from eating food to having symptoms, method of diagnosis, perceived severity of FHS (mild, moderate, severe), how they had treated reactions and if they had called an ambulance or been to hospital because of a reaction.

#### 2.3.1 Eating out behaviours

The survey comprised questions relating to frequency of eating out, reviewing or asking for information when eating out, confidence in verbal and written information provided and how comfortable participants felt about asking staff for information when eating out. The questions were developed through multiple meetings and review by the study team, which included those with FHS, psychologists and clinicians working with patients with FHS and representatives from the Food Standards Agency. Eating out or getting food to take away from a restaurant or other food outlet was measured on a 1 (Never) to 8 (At least once a day) scale. To assess frequency of reviewing information at each stage of eating out, participants were asked: ‘Before deciding where to eat out how often do you check that there is information available that will allow you to identify foods that cause you a bad or unpleasant reaction?’, ‘Before ordering food, how often do you review any available information that allows you to identify foods that will cause you a bad or unpleasant reaction?’ and ‘When eating out how often do you ask a member of staff for information that allows you to identify foods that will cause you a bad or unpleasant reaction?’. Each was responded to using the scale 1 (Never) to 5 (Always). Participants were asked how comfortable they felt asking staff for available information when eating out, measured on a 1 (Not at all uncomfortable) to 4 (Very uncomfortable) scale. Questions relating to confidence in written and verbal information given were measured on a 1 (Not at all confident) to 4 (Very confident) scale.

### 2.4 Analysis

Data were analysed using SPSS version 27. Participants were categorised into one of four FHS groups based on their self‐reported assessment of their reaction to stated foods: food allergy only, food intolerance only, coeliac disease only and multiple hypersensitivities (those who reported more than one type of hypersensitivity, e.g. coeliac disease and intolerance). One‐way ANOVAs were used to examine differences between different FHS groups for the eating out variables (such as frequency of eating out, frequency of asking for information or checking for information before eating out). Independent sample *t*‐tests were carried out to investigate differences across clinical factors (e.g. whether prescribed an adrenaline auto‐injector (AAI), the experience of anaphylaxis, previous history of calling an ambulance, or being admitted to hospital for reaction to FH). Pearson's correlations were run to investigate relationships with continuous variables such as perceived severity of FHS. Multiple linear regression was used to investigate predictors of eating out behaviours. Only variables that were significantly associated with eating out behaviours were entered into the models. When considering clinical variables in any analysis, this related to the participant's first reported food as this was indicated as the food that had the most impact on their lives. All significance levels were set to *p* < 0.05, unless more than 3 comparisons were made (as with the FHS analyses), in which case a Bonferroni correction was applied.

## 3 RESULTS

### 3.1 Participant characteristics

A total of 1019 adults completed the survey. Of these, 89 reported other non‐specified FHSs or did not know what type of FHS they had and were not included in the analysis. Of the 930 adults included, 88% (*n* = 820) were women. The mean age of all participants was 50 years (SD = 16.6), with a range from 18 to 86 years. The majority of adults were from a White background (*n* = 882; 95%). A total of 409 (44%) reported coeliac disease, 216 (23%) reported food intolerance, 170 (18%) reported food allergy and 135 (15%) reported multiple FHS (two or more of these conditions). Participant characteristics can be found in Table 1.

**TABLE 1:** Adult participant characteristics (*n* = 930).

Participant characteristics	Total
	*N* (%)
Gender	
Women	820 (88.2)
Men	110 (11.8)
Ethnicity	
Asian	18 (1.9)
Black	8 (0.9)
Mixed	12 (1.3)
Other	7 (0.8)
White	882 (94.8)
Other long‐term health condition	479 (51.5)
Food allergy	69 (41.1)
Food intolerance	112 (52.1)
Coeliac disease	216 (52.9)
Multiple	82 (60.7)
Symptoms (all reported)	7548
Breathing	1088 (14.4)
Skin	1214 (16.1)
Gastrointestinal	4009 (53.1)
Mouth/ear/throat	821 (10.9)
Other	416 (5.5)
Prescribed an AAI	124 (15.9)
For first food reported	
Experienced anaphylaxis	114 (15.2)
Treatment	
AAI	79 (8.5)
Antihistamines	182 (19.6)
Called an ambulance due to reaction	81 (9.0)
Hospital admission due to reaction	110 (14.1)
Severity	
Mild	109 (11.8)
Moderate	348 (37.5)
Severe	470 (50.7)

Abbreviation: AAI, Adrenaline Auto‐Injector.

A total of 7548 symptoms were reported for 1373 foods. The most common foods were cereals containing gluten (*n* = 615, 45%), milk (*n* = 149, 11%) and peanuts (*n* = 80, 6%). For coeliac disease, the most common food was cereals containing gluten (95%). For food intolerance, it was cereals (30%) followed by milk (20%). For food allergy, the most common was peanut (20%) followed by tree nut (11%). The most common symptoms for coeliac disease and food intolerance were gastrointestinal (81% and 68% respectively). The most common symptoms for food allergy were respiratory (26%), cutaneous (26%), gastrointestinal (20%) and symptoms affecting the mouth, throat, or ears (20%). Most participants with a food allergy (79%) or coeliac disease (98%) had received a medical diagnosis of their FHS, whereas only 42% of those reporting food intolerance had received a medical diagnosis.

#### 3.1.1 Risk assessment by the different Food hypersensitivity groups when eating out

Half of the overall sample (*n* = 549; 56%) reported that they eat out or get food to take away from a restaurant or other food outlet once a month or more, with a third (*n* = 338, 35%) reporting that they eat out less than once a month and only 9% said never. There were significant differences in how often adults with different FHSs ate out, *F* (3,892) = 5.41, *p* = 0.001. Those with allergy reported eating out once a month or fortnight, and this was significantly more often (mean = 3.4, SD = 1.5) than those with food intolerance (mean = 3.0, SD = 1.3), those with coeliac disease (mean = 2.9, SD = 1.2) and those with multiple FHS (mean = 3.0, SD = 1.5) who on average ate out around once a month (all *p* values for post hocs <0.008).

Most adults (79%) checked information before deciding where to eat out always or most of the time; however, adults with multiple FHS or coeliac disease checked information significantly more frequently than those with food allergy or intolerance (all *p* values for post hocs <0.008) (Table 2). Most adults (84%) reviewed information before ordering food always or most of the time; however, again there were differences across FHS groups. Adults with food intolerance reported that they review available information before ordering food significantly less often (about half or most of the time) than adults with other FHS, who review this either always or most of the time (all *p* values for post hocs <0.001) (Table 2).

**TABLE 2:** Means (and standard deviations) for reviewing information at each stage of eating out across different food hypersensitive groups.

Risk assessment behaviour	Food allergy	Food intolerance	Coeliac disease	Multiple FHS	*F* (df)
	*M* (SD)	*M* (SD)	*M* (SD)	*M* (SD)	
Check information when choosing venue	4.2 (1.3)	3.3 (1.6)	4.8 (0.6)	4.6 (0.9)	68.4*** (3,808)
Review information before ordering	4.4 (1.0)	3.6 (1.6)	4.9 (0.4)	4.8 (0.8)	70.70*** (3,806)
Ask staff for information when ordering	3.9 (1.4)	2.9 (1.6)	4.7 (0.7)	4.5 (1.0)	108.2*** (3,809)

***p < 0.001.

Three quarters of adults (74%) asked the staff for information that allowed them to identify foods that would cause them a bad or unpleasant reaction before ordering always or most of the time. However, adults with coeliac disease and multiple FHS on average reported asking staff significantly more often than those with food allergy or food intolerance (all *p* values for post hocs <0.001) (Table 2).

Over half of all participants (*n* = 542; 61%) were very or fairly comfortable in asking staff for information when eating out because of a concern about experiencing a reaction. There were no significant differences across the FHS groups (63% of the food allergy, 64% of the food intolerance, 61% of the coeliac disease and 59% of the multiple FHS group reporting being very or fairly comfortable).

#### 3.1.2 Risk assessment when eating out across different clinical variables

Frequency of checking information before deciding where to eat, reviewing information before ordering food, or asking staff for information varied depending on clinical variables related to the severity of the reaction. Information was checked or staff were asked significantly more often if participants reported a medical rather than self‐diagnosis, had another long‐term health condition, had been prescribed an AAI, had a previous anaphylactic reaction, called an ambulance for a reaction, or had been admitted to hospital due to a reaction (all *p* values < 0.001; see Table 3). Greater self‐reported severity of FHS also significantly correlated with more frequent checking before deciding where to eat (*r* = 0.46, *p* < 0.001), checking before ordering (*r* = 0.48, *p* < 0.001) and asking staff (*r* = 0.49, *p* < 0.001).

**TABLE 3:** Means (and standard deviations) for reviewing information at each stage of eating out according to clinical factors.

Clinical variable	Check information when choosing venue		*t* (df)	Review information before ordering		*t* (df)	Ask staff for information when ordering		*t* (df)
	*M* (SD)	*M* (SD)		*M* (SD)	*M* (SD)		*M* (SD)	*M* (SD)	
	Yes	No		Yes	No		Yes	No	
Medical diagnosis	4.5 (1.0)	3.1 (1.7)	11.50** (247.3)	4.7 (0.9)	3.5 (1.6)	10.38** (239.9)	4.4 (1.1)	2.7 (1.6)	13.68** (262.0)
Long‐term condition	4.4 (1.2)	4.0 (1.4)	4.07** (856.6)	4.5 (1.1)	4.2 (1.3)	3.31** (854.9)	4.2 (1.3)	3.8 (1.5)	3.66** (863.2)
Prescribed an AAI	4.6 (0.9)	4.1 (1.4)	4.60** (173.9)	4.8 (0.5)	4.3 (1.3)	7.37** (346.8)	4.5 (0.9)	3.9 (1.5)	5.81** (191.1)
Experience of anaphylaxis	4.6 (1.0)	4.1 (1.4)	4.20** (161.2)	4.8 (0.5)	4.3 (1.3)	7.29** (297.9)	4.5 (0.9)	3.9 (1.5)	5.89** (194.5)
Called an ambulance	4.6 (1.0)	4.1 (1.4)	3.90** (97.1)	4.8 (0.7)	4.3 (1.3)	4.98** (125.0)	4.6 (0.9)	3.9 (1.5)	5.94** (112.3)
Admitted to hospital	4.8 (0.7)	4.1 (1.4)	7.89** (213.9)	4.9 (0.4)	4.3 (1.3)	9.48** (424.5)	4.7 (0.7)	3.9 (1.5)	8.81** (226.9)

Abbreviation: AAI, Adrenaline Auto‐Injector.

**p < 0.001.

#### 3.1.3 Predictors of risk assessment behaviour when eating out

Variables that were significantly associated with these risk assessment behaviours were entered into regression models (Table 4). For checking before deciding where to eat out, variables significantly predicted 27% of the variance. Food hypersensitivity group, severity of FHS and type of diagnosis (medical vs. self) significantly predicted frequency of checking, with severity being the strongest predictor (all *p* < 0.001). For reviewing information before ordering, variables significantly predicted 27% of the variance. Again, FHS group, severity of FHS and type of diagnosis (medical vs. self) significantly predicted frequency of checking, with severity being the strongest predictor (all *p* < 0.001). For asking staff before ordering, variables significantly predicted 35% of the variance. Again, FHS group, severity of FHS and type of diagnosis (medical vs. self) significantly predicted frequency of checking, with severity being the strongest predictor (all *p* < 0.001) (Table 4).

**TABLE 4:** Regression models for reviewing information when eating out.

Predictor variable	Check information when choosing venue		Review information before ordering		Ask staff for information when ordering	
	*β*	CI	*β*	CI	*β*	CI
FHS group	0.20***	0.1 –0.35	0.19***	0.14–0.30	0.26***	0.27–0.46
Severity	0.29***	0.39–0.62	0.32***	0.39–0.60	0.31***	0.47–0.72
Medical diagnosis	−0.25***	−0.88 to −0.51	−0.21***	−0.68 to −0.36	−0.26***	−0.96 to −0.59
Long‐term condition	−0.01	−0.16–0.14	0.03	−0.06–0.20	−0.02	−0.09–0.22
Prescribed an AAI	−0.03	−0.40–0.21	−0.06	−0.48–0.07	−0.07	−0.60–0.03
Experience of anaphylaxis	−0.01	−0.26–0.20	−0.01	−0.23–0.18	−0.004	−0.25–0.22
Called an ambulance	0.01	−0.33–0.44	0.03	−0.25–0.43	−0.01	−0.42–0.37
Admitted to hospital	−0.08	−0.62–0.06	−0.05	−0.45–0.15	−0.07	−0.62–0.08
*R* ^2^; Adj *R* ^2^	0.53; 0.27		0.52; 0.27		0.60; 0.35	
*F* (df)	37.68 (8,767)***		35.72 (8,768)***		52.41 (8,771)***	

Abbreviation: AAI, Adrenaline Auto‐Injector.

***p < 0.001.

#### 3.1.4 Confidence in information when eating out

Over half of the adults (*n* = 545; 62%) reported feeling very or fairly confident in the written information provided to allow them to identify foods that would cause a bad or unpleasant reaction. However, there were some differences across FHS groups (*F* (3) = 3.85, *p* = 0.01, ηp^2^ = 0.01). Those with food intolerance (mean = 2.9, SD = 0.7) were significantly more confident in written information than those with multiple FHS (mean = 2.6, SD = 0.8; *p* < 0.008).

Only 43% (*n* = 384) of adults were very or fairly confident about verbal allergen information provided by eating out venues, and 36% (*n* = 321) not very or not at all confident in the information provided. There were significant differences across the different FHS groups (*F* (3) = 6.52, *p* < 0.001, ηp^2^ = 0.03). Those with food intolerance (mean = 2.7, SD = 0.8) were significantly more confident in verbal information provided by staff compared with participants with food allergy (2.4, SD = 0.9), coeliac disease (mean = 2.5, SD = 0.8), or multiple FHS (mean = 2.3, SD = 0.8; all *p*s < 0.008).

Those who reported a previous anaphylactic reaction, called an ambulance, or been hospitalised were significantly less confident in verbal or written information (see Table 5). In addition, those who had been prescribed an AAI or had a medical rather than a self‐diagnosis were also less confident in verbal information provided (see Table 5). These variables were entered into regression models. Although the models were significant, only 1% of variance was explained and having a previous anaphylactic reaction was the only significant predictor of confidence in written or verbal information.

**TABLE 5:** Means and standard deviations for confidence in information provided when eating out according to clinical factors.

Clinical variable	Confidence in written information		*t* (df)	Confidence in verbal information		*t* (df)
	*M* (SD)	*M* (SD)		*M* (SD)	*M* (SD)	
	Yes	No		Yes	No	
Medical diagnosis	2.8 (0.8)	2.9 (0.7)	−1.10 (224.28)	2.4 (0.8)	2.7 (0.7)	−3.10** (190.17)
Prescribed an AAI	2.7 (0.8)	2.8 (0.7)	−1.56 (104.63)	2.2 (0.9)	32.5(0.8)	−3.21*** (632)
Experience of anaphylaxis	2.6 (0.8)	2.8 (0.7)	−2.09* (107.26)	2.2 (0.8)	2.5 (0.7)	−3.76*** (615)
Called an ambulance	2.5 (0.8)	2.8 (0.7)	−2.68** (67.85)	2.1 (0.9)	2.5 (0.8)	−4.01*** (635)
Admitted to hospital	2.6 (0.9)	2.8 (0.7)	−2.82** (92.8)	2.2 (0.9)	2.5 (0.8)	−3.78*** (634)

Abbreviation: AAI, Adrenaline Auto‐Injector.

*p < 0.05; **p < 0.01; **p < 0.001.

## 4 DISCUSSION

The aim of this study was to explore the differences in experiences and risk assessment practices by adults with different FHS when eating out. There were significant differences in risk assessment behaviours across FHS groups with those with food intolerance conducting risk assessments less often than the other FHS groups. Adults conducted risk assessments more frequently if they had a medical diagnosis, perceived their FHS as severe, had another long‐term health condition, had prescribed an AAI, had experienced an anaphylactic reaction, called an ambulance for their reaction, or been hospitalised for their reaction. In the FHS group, perceived severity of FHS and having a medical diagnosis consistently predicted risk assessment behaviour.

Differences in the frequency with which information is checked when eating out may be due to a fear of the consequences of having an accidental reaction. Those with intolerance checked less often and this type of FHS is typically associated with symptoms which are not life‐threatening. Half of those with food intolerance also did not have a medical diagnosis. Across the whole sample, those with a self‐diagnosis also checked less often. This may indicate a lack of knowledge or lack of perceived seriousness of their FHS. The severity of FHS as reported by the adults was a consistent significant predictor of behaviour, which supports this theory, as do the significant associations with being prescribed an AAI. Past experience of a severe reaction, calling an ambulance, or going to hospital due to a reaction were also associated with more frequent checking. This experience may be a prompt for future more vigilant behaviour; however, the frequent checking of those with severe reactions may place a greater burden on individuals and may impair their QoL and mental health.^12^ Therefore, ensuring that clear and accurate information for those with FHS when eating out would be beneficial. Our data also highlights the importance of a formal medical diagnosis of FHS on risk assessment, as those with a diagnosis were more vigilant prior to consumption of foods and drinks.

The majority of adults across all FHS groups reported feeling very or fairly comfortable about asking for information when eating out; however, there were differences regarding their confidence in the information provided. Adults with FHS were more confident in written compared to verbal information and those with food intolerance were more confident compared to those with food allergy, coeliac disease and multiple FHS. These differences indicate that the type of FHS is a significant factor when considering confidence and it may be that perception of severity of the consequences of eating something that could cause symptoms is again a key factor. Those with severe reactions may be less trusting in information, possibly because the risk is greater and consequences could be more severe. This is also supported by the results that adults with previous experience of anaphylaxis, hospitalisation or those who were prescribed an AAI were significantly less confident in both written and verbal information provided when eating out. As Begen et al.^13^ found when exploring issues in caregiver interviews, these results suggest that to cater for all those with FHS and varying severities of FHS, a variety of strategies are needed by food outlets. As confidence was reported to be highest in written information, making this information more easily available could help adults manage their FHS whilst eating out. However, these results also demonstrate a need for improved staff training and improved verbal communication between consumers and staff in eating out establishments in order for trust in this information to be increased.^14^ Clinicians should also be aware that the type of FHS, patient‐perceived severity and past experience of reactions are associated with risk assessment behaviours when eating out. These factors should be considered when providing clinical advice and emergency plans.

Although this is the first large‐scale quantitative study to our knowledge to consider risk assessment practices of adults with different types of FHS when eating out, there are some limitations to consider. A quantitative online survey approach allowed for a large community sample to be collected; however, it relies on self‐reporting of diagnosis. While the majority with food allergy or coeliac disease stated a medical diagnosis and reported foods, symptoms and time between ingestion and symptoms which aligned with their FHS, less than half of those with food intolerance reported a medical diagnosis. This is not surprising given the lack of diagnostic tests for intolerance. It is also important to note that the belief one has a FHS and needs to avoid food will have an impact on eating out behaviours, whether that FHS is medically confirmed or not.^11^

In this study, nearly 90% of the participants were women. This could in part be because the incidence of food allergy, food intolerance and coeliac disease is reported to be higher in females than males.^15^, ^16^, ^17^ However, in general, response rates to online surveys are often higher for women than men.^18^ Most of the sample was also from a White ethnic background. We cannot say whether the findings reported here would be similar for people of other genders or ethnicities, or those who do not have access to the Internet or who are not proficient in English. Their eating out experiences may be different and future research should aim to collect data from across a broader demographic. The regression models accounted for up to a third of variance in risk assessment behaviour. Therefore, it is important for future studies to explore other factors that might have an impact. These could be individual factors such as self‐efficacy for food allergy management or situational factors such as the type of meal being ordered or the type of eating out establishment. This information would help clinicians, food establishments and patients develop ways to support safe eating practices.

## 5 CONCLUSIONS

This research has shown that there are differences in risk assessment behaviours of adults when eating out based on the type of FHS, severity of the reaction, and previous experience of severe reactions resulting in anaphylaxis or hospitalisation. This has important implications for the food industry and for the advice clinicians give their patients. There are differences in how individuals with different types of FHS approach eating out and there is a need for an awareness of this, and for food establishments to consider and implement ways they can cater to the multiple needs of those managing different FHSs. Those who suffer from more severe reactions may be more likely to check information but also have the least confidence in the information provided. Therefore, training of staff in how to discuss dietary requirements, and clear provision of written and verbal allergen information at eating out establishments is needed to improve confidence for those with FHS. Tailored advice from clinicians depending on the type of FHS, taking into account the patient's perceived severity of the FHS and previous experience of a reaction, is also recommended.

## AUTHOR CONTRIBUTIONS

Funding for the study was provided by Rebecca C. Knibb. The survey design was carried out by a working group involving all authors. Recruitment was led by Rebecca C. Knibb, Lily Hawkins and Cassandra Screti. Data analysis was carried out by Lily Hawkins and Rebecca C. Knibb. The manuscript preparation was led by Rebecca C. Knibb and Lily Hawkins. All authors approved the final draft for publication.

## CONFLICT OF INTEREST STATEMENT

RCK declares research funding from the Food Standards Agency, NIHR RfPB, Aimmune, Novartis, National Peanut Board. MHG declares research funding from the Food Standards Agency and UKRI via Queen's University Belfast and the University of Bath, lecture fees from UK universities, advisory fees from the Food Standards Agency and Safefood, and consultancy and training fees from UK food businesses. CJ declares research funding from the Food Standards Agency, NIHR RfPB, Aimmune, Novartis, National Peanut Board and honoraria from NIHR RfPB, Nutricia and Allergy UK. MTK is Chair of Equality Diversity and Inclusion Working Group, BSACI. He has received research grants from NIHR, GCRF, University of Birmingham and MRC CiC. His department at UHB has received educational grants from ALK Abello, Thermofisher, MEDA and other pharmaceutical companies for the annual PracticAllergy course. GdT reports grants from National Institute of Allergy and Infectious Diseases (NIAID, NIH), Food Allergy & Research Education (FARE), MRC & Asthma UK Centre, Action Medical Research and National Peanut Board. Scientific Advisory Board member Aimmune and Novartis. Investigator on pharma‐sponsored allergy studies (Aimmune, DBV Technologies, Novartis).

## Data Availability

Data available on request due to privacy/ethical restrictions.
